# Graphene quantum dots as nanoprobes for fluorescent detection of propofol in emulsions

**DOI:** 10.1098/rsos.181753

**Published:** 2019-01-23

**Authors:** Juanjuan Diao, Tingting Wang, Li Li

**Affiliations:** Pharmacy College, Xinjiang Medical University, Urumqi 830011, People's Republic of China

**Keywords:** graphene quantum dots, propofol, fluorescence, emulsions

## Abstract

We report a new fluorescent detection method for propofol based on graphene quantum dots (GQDs). Citric acid (CA) was selected as the carbon precursor, and fluorescent GQDs were prepared by carbonizing CA. The product, which efficiently quenched the fluorescence of GQDs, could be obtained through the oxidation of propofol in the presence of horseradish peroxidase and hydrogen peroxide. The fluorescence intensity ratio of GQDs (*F*/*F*_0_) was positively correlated with the concentration of propofol, which ranged within 5.34–89.07 mg l^−1^, the limit of detection was 0.5 mg l^−1^ and the limit of quantity was 5.34 mg l^−1^. The developed fluorescence method reported in the present study is simple, sensitive, reproducible, and can serve in determining propofol contents in emulsions.

## 1. Introduction

Graphene quantum dots (GQDs), emerged as novel materials in the recent decade, and have demonstrated superiority in numerous privileged properties [1]. GQDs are considered superior due to facile preparation methodologies, low toxicity, high luminescent properties and high photostability against photobleaching and blinking, which have attracted substantial attention [2–7]. GQDs have been widely employed in many applications such as the detection of proteins [8], nucleic acids [9,10], inorganic ions [11,12], small organic molecules [13,14] and biological imaging [15,16].

As a new potential tool in analytical chemistry, most of the sensors and biosensors were developed by conventional, doped or functionalized GQDs, in order to improve the sensitivity, selectivity and specificity of fluorescent detection [17]. Based on such attractive features, GQD nanoprobes have been employed in the detection of Fe^3+^ [12], Hg^2+^ [18], Cl^−^ [11], trinitrotoluene, hydroquinone [13] and glucose [19]. GQDs have also been used in the fluorescence detection of phenols, which exhibited an enhanced detection sensitivity level (10^−8^–10^−10^ mol l^−1^). A recent study demonstrated an effective GQD-based approach, and has been reported to be a sensitive method for the detection of dihydroxybenzene (DHB) [20]. Ruiling Sun *et al*. found GQDs had the potential to be applied for analysing the contents of phenol in industrial water using the resonance light scattering method [21]. In food packaging, a detection method based on GQDs has been reported as an efficient approach for the analysis of bisphenol A [22]. Furthermore, GQDs coated with molecularly imprinted polymer have also been reported for the determination of 4-nitrophenol in water samples [23]. The potential use of GQDs as optical nanoprobes for the determination of phenolic contents in olive oil and bifunctional nanoprobes has been applied in a sensing platform for the non-enzymatic photoluminescence detection of hydroquinone [13,24]. However, these methods are mainly used for the detection of water-soluble phenolic compounds, a GQDs-based detection method for fat-soluble phenolic compounds has not been reported to date. This experiment was to study the detection method for a fat-soluble phenolic anaesthetic propofol (2,6-diisopropylphenol) based on GQDs.

In this study, we aimed to investigate the application of GQDs for the determination of propofol in emulsions. Propofol is a short-acting intravenous anaesthetic agent frequently used for anaesthetic induction, maintenance and sedation [25]. Propofol metabolizes quickly in human beings and requires continuous infusion. Owing to individual differences, propofol metabolism is highly variable in nature, which motivated our laboratory to develop a simple detection method for monitoring propofol in clinical settings [26–28].

**Scheme 1.**
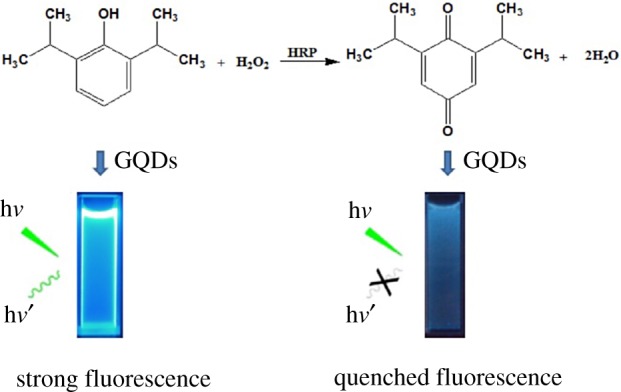
Schematic illustration of propofol detection.

To date, propofol detection methods include, gas chromatography–mass spectrometry (GC–MS) [29], liquid chromatography–mass spectrometry (LC–MS) [30], high-performance liquid chromatography (HPLC) [31], capillary electrophoresis [32], fluorophotometry [33] and UV–Vis spectrophotometry [34–36]. Fluorophotometry is a simple, sensitive and easily detectable technique [37]. Thus, establishing a rapid method to detect propofol is important for the safety and effectiveness of clinical anaesthetics. In the present study, we report a method for determining propofol in emulsions based on GQDs and fluorescent spectrophotometry. GQDs have been employed as novel nanofluorescence probes designed to detect propofol in emulsion with sensitivity and selectivity. The proposed simple and environment-friendly approach provides a novel method to probe the practical application of GQDs in propofol emulsions. The presently developed method was based on the oxidative reaction of propofol in the presence of hydrogen peroxide (H_2_O_2_) and horseradish peroxidase (HRP) to obtain the corresponding benzoquinone, which could quench the fluorescence of GQDs efficiently, as depicted in scheme 1.

## 2. Material and methods

### 2.1. Reagents and standards

Propofol standard (greater than 99.8%) was purchased from the National Institute for Food and Drugs Control in China. Propofol injection (1% w/v, 10 mg ml^−1^) was purchased from Guori Pharmaceutical Co., Ltd (Sichuan, China). Citric acid (CA, 99%) and NaOH were obtained from Shengao Chemical Reagent Co., Ltd. Absolute alcohol and glycerol were purchased from Tianjin Fuyu Chemical Co., Ltd (Tianjin, China). H_2_O_2_ (30%, v/v) was obtained from Tianjin Beilian Chemicals Co., Ltd (Tianjin, China). HRP (greater than 150 units mg^−1^) was purchased from Yuanye Biological Technology Co., Ltd (Shanghai, China). Egg yolk lecithin was purchased from Shanghai Lanji Science and Technology development Co., Ltd (Shanghai, China). Soya bean oil was purchased from Jiangxi Yipusheng Pharmaceuticals Co., Ltd (Nanchang, China). All reagents were of analytical grade and used without additional purification.

### 2.2. Instrumentation

UV–Vis absorption spectra were obtained using a Shimadzu UV–2550 spectrophotometer (Shimadzu, Japan). A Shimadzu RF-5301 fluorescence spectrometer (Shimadzu, Japan) was used to record the fluorescence emission spectra of GQDs using a quartz cuvette cell (path length: 1 cm). An IRPrestige-21 FT-IR spectrometer (Shimadzu, Japan) was used to obtain the Fourier transform infrared (FTIR) spectra. Transmission electron microscopy (TEM) was conducted through a JEM-1230 transmission electron microscope (JEOL, Japan) operating at 200 kV. HPLC was carried out using chromatography apparatus (Shimadzu, Japan) equipped with an LC-20AT pump, SPD-M20A diode array detector, CBM-20A controller and CTO-20A column oven. A Shim-pack VP-ODS C18 column (5 µm, 250 × 4.6 mm internal diameter; Shimadzu) was used for reserve phase HPLC. The pH was measured with a PHSJ-3F digital pH-meter (Leici, China).

### 2.3. Preparation of graphene quantum dots

GQDs were synthesized by incompletely carbonized CA, as previously described by Dong *et al.* [7]. Briefly, 2.0 g of CA was placed in a round bottom flask, which was subsequently heated to 210°C in a heating mantle. After 50 min, when the liquid CA turned to pale-yellow and then to orange, 65 ml of 10 mg ml^−1^ NaOH was quickly added with stirring to adjust the pH of the mixed solution to 7.0, and the solution was stored at 4°C until used.

### 2.4. Analysis of propofol in the presence of horseradish peroxidase/hydrogen peroxide

For the propofol assay, 1055 mg l^−1^ propofol solution was diluted with 96% ethanol to obtain different concentrations of propofol solutions. Then, 96% ethanol was used for dilution of the propofol emulsion injection, in order to obtain higher solubility of propofol in real sample emulsion.

A 0.2 mol l^−1^ phosphate buffer was prepared from potassium dihydrogen phosphate, and adjusted to pH 7.4 by adding 0.2 mol l^−1^ sodium hydroxide solution. Then, H_2_O_2_ and HRP were dissolved in and diluted with buffer solution, respectively [33]. Next, 0.5 ml of 20 mmol l^−1^ H_2_O_2_ was added, followed by 0.5 ml of propofol solution with different concentrations and 0.5 ml of 2.5 mg ml^−1^ HRP. The mixture was heated at 40°C for 5 min. Then, 1.5 ml of 0.5% GQDs were added to a final volume of 3 ml. The obtained solution was shaken gently and stored for half an hour at room temperature before performing the fluorescence spectrophotometry [20]. The fluorescence quenching spectra were recorded under the following conditions: an excitation wavelength of 365 nm, a maximum emission of 460 nm, and slit widths of 5.0 and 5.0 nm for excitation and emission, respectively.

## 3. Results and discussion

### 3.1. Spectral characterization of graphene quantum dots

The incompletely carbonized CA was used for surface-passivation of GQDs. After moderate pyrolysis, CA could be partially carbonized to form GQDs. As GQDs contained abundant small sp^2^ clusters which were non-uniform in size and poorly passivated [7], and thus were possibly isolated within the sp^3^ C–O matrix, the GQDs exhibited an excitation-dependent photoluminescent (PL) activity.

**Figure 1.**
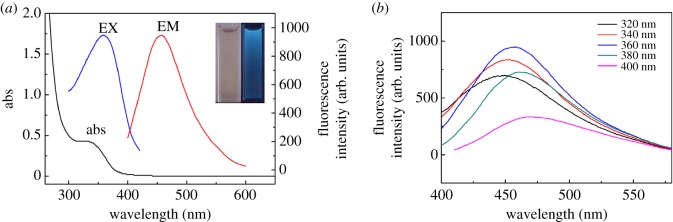
(*a*) UV–Vis absorption spectrum (black line) and fluorescence emission spectrum (coloured line) of GQDs. (*b*) Emission spectra of the GQDs with excitation of different wavelengths. Inset: photographs of the GQDs solution taken under visible light (left) and 360 nm of UV light (right).

The PL emission and UV–Vis absorption spectra of the GQDs solution are depicted in figure 1*a*. The UV–Vis absorption spectrum of the prepared GQDs showed a shoulder peak at 340 nm (figure 1*a*). GQDs revealed fluorescence properties, as shown by the apparent absorption band at 365 nm, corresponding to the excitation spectra and displayed the fluorescence intensity (maximum emission) at 460 nm. Based on UV–Vis spectral results, it is possible to infer that CA polymerization changed the material structure to attain fluorescence characteristics.

In order to further characterize the optical characteristics of GQDs, a comprehensive photoluminescence study was conducted at different excitation wavelengths (figure 1*b*). The prepared GQDs solution also emitted blue light upon excitation at 320–400 nm. Maximum emission was achieved at 360 nm of excitation, as shown in figure 1*b*. GQDs exhibited an excitation-dependent PL behaviour.

### 3.2. Fourier transform infrared spectral characteristics and transmission electron microscopy image of graphene quantum dots

**Figure 2.**
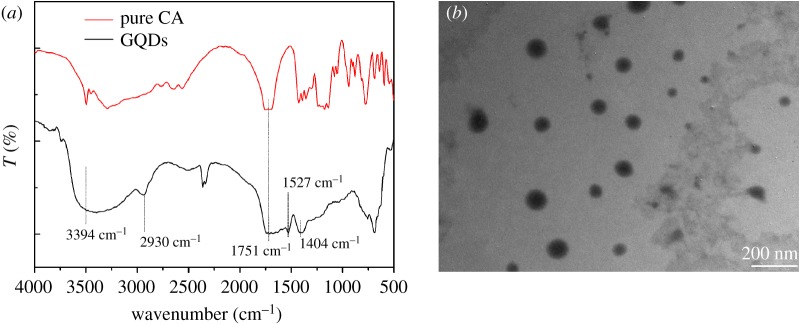
(*a*) FTIR spectra of citric acid and GQDs. (*b*) TEM image of GQDs.

FTIR spectra were recorded to investigate and characterize the chemical bonding states of GQDs (figure 2*a*). The FTIR spectrum of the GQDs revealed intense absorption bands. The CA and GQDs revealed –OH stretching vibrations at 3394 cm^−1^ and –C = O stretching vibrations at 1751 cm^−1^, but the GQDs revealed –CH_2_ stretching vibrations at 2930 cm^−1^ and C = C stretching vibrations at 1527 cm^−1^. These results indicate the dehydration of CA during hydrothermal polymerization. The peak appeared at 1404 cm^−1^, due to the C–O bond stretching. CA was completely carbonized to form graphene oxide (GO). The C–H bond of GO was not observed in the FTIR spectra, but stretching vibration of the C–O–C group appeared at 1012 cm^−1^. Under FTIR characterization, GQDs revealed the absorption for the C–H bond, but without the absorption of the C–O–C functional group, which suggests that GQDs were the products of the incomplete carbonation of CA.

The particle size of GQDs was determined, and the sample was analysed by TEM. The TEM image of GQDs are shown in figure 2*b*. The diameter of GQDs ranged within 10–85 nm, and most of the fractions were large particles with inhomogeneous sizes, indicating that CA carbonation altered the particle size (figure 2*b*). Shujun Wang *et al*. suggested that the PL properties of GQDs are possibly due to the competition between large particles of broad size distribution and a narrow size range (0.7–1.0 nm) with the small particles. A fraction of small uniformly sized GQDs (0.7–1.0 nm) contributes to the excitation-independent fluorescence, whereas the fraction of large inhomogeneously sized GQD (ranging from 35 to 85 nm) structures dominated the excitation-dependent fluorescence [38]. It was inferred that in the present investigation, the large fraction of inhomogeneously sized GQDs was dominant in quantity. Hence, an excitation-dependent photoluminescence was observed.

### 3.3. Optimization of experimental parameters for propofol detection

The fluorescence quenching of GQDs occurred due to the oxidation of propofol by H_2_O_2_ and HRP to form benzoquinone. This has been reported to be an efficient electron acceptor that could mediate the electron transfer of the excited luminescence material, which finally leads to the effective fluorescence quenching of GQDs [20]. It is noteworthy that H_2_O_2_, HRP and the propofol solution did not induce the fluorescence quenching of GQDs. However, the product that reacted with H_2_O_2_, HRP and propofol was observed to induce GQDs fluorescence quenching.

**Figure 3.**
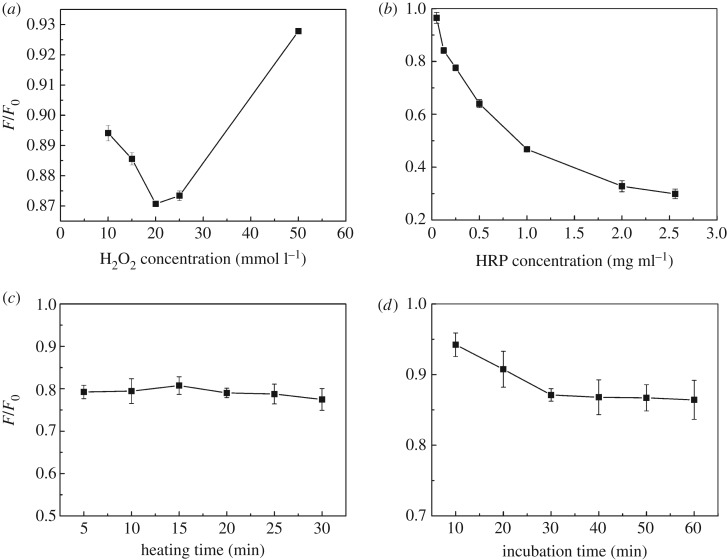
Effect of concentrations of H_2_O_2_ and HRP, heating time and incubation time on *F/F*_0_. (*a*) Effect of H_2_O_2_ concentration on fluorescence. (*b*) Effect of HRP concentration on fluorescence. (*c*) Effect of heating time on fluorescence. (*d*) Effect of incubation time on fluorescence.

In order to improve the analytical characteristics, the developed method was optimized against several parameters such as the concentration of H_2_O_2_ and HRP, and the relationship between incubation time and GQDs fluorescence intensity. In order to accomplish this, several concentrations of H_2_O_2_ were prepared. When the HRP concentration was 2.27 mg ml^−1^, the H_2_O_2_ concentration was studied within the range of 10–50 mmol l^−1^. The quenched fluorescence spectrum of each H_2_O_2_ solution was measured. Fluorescence quenching was highest during the reaction with 20 mmol l^−1^ of H_2_O_2_ (figure 3*a*), which was selected for the subsequent trials.

With HRP as the catalyst, enzyme concentrations ranging within 0.05–2.50 mg ml^−1^ were investigated when the H_2_O_2_ concentration was 20 mmol l^−1^. The high enzyme concentration resulted in high quenching (figure 3*b*). Further studies were conducted using the HRP concentration (2.50 mg ml^−1^) as optimal.

Heating duration affects both the reactant formation and enzyme activity. The influence of heating time was studied within the range of 5–30 min at 40°C, and was found to slightly affect the fluorescence (figure 3*c*). A heating duration of 5 min at 40°C was chosen as favourable heating conditions. The relationship between incubation time and GQDs fluorescence intensity was investigated, as shown in figure 3*d*. GQDs fluorescence intensity was quenched upon the addition of propofol, and the incubation time was studied within the range of 10–60 min. The fluorescence quenching produced upon the addition of GQDs became stable after 30 min of incubation time, and this slightly decreased thereafter. After the optimization of the relationship between incubation time and GQDs, further experiments were conducted using 30 min as the incubation duration.

### 3.4. Quantitative detection of propofol

**Figure 4.**
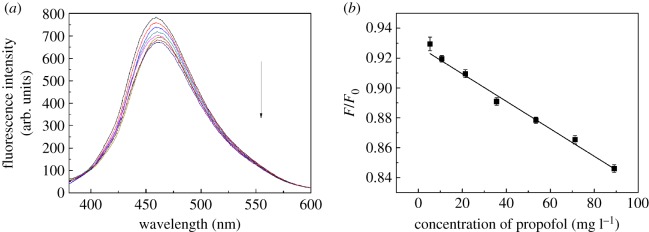
The relationship between fluorescence quenching and the different concentration of propofol is shown (from top: 0, 5.34, 10.69, 21.38, 35.63, 53.44, 71.26 and 89.07 mg l^−1^).

Using the above-optimized conditions, the quenching effect of propofol on GQDs fluorescence intensity was further studied. As shown in figure 4, the fluorescence spectra of GQDs under different propofol concentrations were determined with the presence of 20 mmol l^−1^ of H_2_O_2_ and 2.5 mg ml^−1^ of HRP. It was observed that with the increase in propofol concentrations, the quenching amount also gradually increased. A good linear correlation was achieved within the range of 5.34–89.07 mg l^−1^, *R*^2^ = 0.9932 (figure 4). The linear regression equation was *F*/*F*_0_ = 0.9620−0.0013 *C*_(propofol)_ (mg l^−1^). The limit of detection (LOD) was 0.5 mg l^−1^ and the limit of quantity (LOQ) was 5.34 mg l^−1^. The data show the average of three independent experiments (*n* = 3). In table 1, the detection limits and linear ranges of the experiments are consistent with previous reports. Results using the standard addition methods are shown in table 2. The average recoveries from 112.6% to 118.4% were observed in real samples, and the relative standard deviation (RSD) was less than 0.95%, indicating the potential of the GQDs and HRP system for detecting propofol in emulsion injection samples.

**Table 1.** Comparison of the present method with the reported methods for the determination of propofol.

methods	linear range (mg l^−1^)	LOD (mg l^−1^)	refs.
HPLC	0.05–10	0.005	[31]
GC–MS	0.025–5	0.01	[32]
HPLC–MS/MS	0.001–0.1	0.001	[33]
capillary electrophoresis	0.1–16	0.1	[34]
fluorophotometry	4–243	1.3	[35]
UV–Vis	3.0–18	0.65	[36]
graphene quantum dots	5.34–89.07	0.5	this work

**Table 2.** Analysis of propofol in emulsion injection samples.

Leweijing propofol injection emulsion (mg l^−1^)	propofol			
	added	found	recovery (%)	RSD (*n* = 3, %)
sample 1	21.38	24.04 ± 0.23	112.6	0.95
sample 2	26.36	30.95 ± 0.13	117.4	0.41
sample 3	31.35	37.14 ± 0.28	118.4	0.75

### 3.5. Sample determination

Three batches of Leweijing propofol injection emulsion were obtained. The samples were dissolved in 96% ethanol. The lipophilic analyte propofol in the emulsion was completely dissolved in 96% ethanol. The determination of samples from each batch was repeated three times. The average contents of the three batches of propofol emulsion were determined as 9.84, 9.78 and 9.82 mg ml^−1^, respectively (RSD < 5.0%). Recoveries of propofol within the range of 112.6–118.4% suggest that the method produced satisfactory results for practical applications, although the sensitivity of the method needs to be further improved. This method can be used for actual propofol determination in emulsions.

### 3.6. Interference study

**Figure 5.**
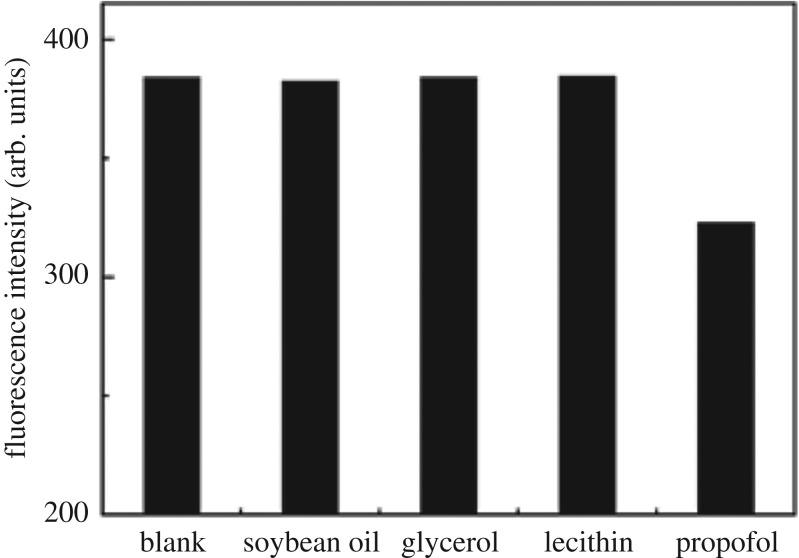
The FL response of the sensing system in the presence of soybean oil, glycerol, lecithin and propofol.

Propofol injectable emulsion contains propofol in a 1% (w/v) oil-in-water sterilized emulsion, which also contains 100 mg ml^−1^ soybean oil, 22.5 mg ml^−1^ glycerol, 12 mg ml^−1^ lecithin and sodium hydroxide for pH adjustment. The pH of the emulsion was 7.0–8.5. The excipients in the injection emulsion may interfere with the determination of GQDs. The influence of some excipients, including soya bean oil, glycerol and lecithin, were investigated (figure 5). The highest concentration was considered as the tolerance of each coexistence substrate, with a relative error less than ±5%. The result indicated that the excipients did not interfere with the determination, which further signifies the high selectivity of the proposed method for determining propofol in emulsions.

### 3.7. High-performance liquid chromatography method validation

The HPLC analysis was conducted using a Shimadzu chromatographic system, which consists of a LC-20AT pump with an automatic injector, and a SPD-M20A diode array detector. Separation was achieved with the Shim-pack VP-ODS C18 column (5 µm, 250 × 4.6 mm internal diameter; Shimadzu) maintained at 40°C. The chromatographic run was performed at 1 ml min^−1^ with a gradient elution of a mobile phase, which consisted of Solution A (0.02 mol l^−1^ of NaH_2_PO_4_ buffer, pH 3.0) and Solution B (Acetonitrile). The percentage of organic modifier (B) was as follows in a linear change: 0 min, 40%; 22 min, 40%; 38 min, 70%; 40 min, 70%; 41 min, 90%; 45 min, 90%; 46 min, 40%. The injection volume was 20 µl and total analysis run time was 46 min. The detection wavelength was 275 nm. The propofol injection emulsion was diluted with anhydrous ethanol as a test solution. The propofol standard solution was also diluted with anhydrous ethanol, and the concentration was maintained at 1.0 mg ml^−1^. This was used as an external standard for quantitative analysis.

A comparison of the propofol determination that resulted from samples using the GQDs method and HPLC method is presented in table 3. After conducting the Wilcoxon signed-rank test (inspection level: *α* = 0.05), the concentrations of propofol in the emulsion obtained by the two types of measurement methods revealed no significant differences (*p* > 0.05).

**Table 3.** Concentrations of propofol in emulsion using the GQD and HPLC methods.

Leweijing propofol injection emulsion	measured concentration (mg ml^−1^)	
	GQDs method	HPLC method
sample 1	9.84 ± 0.15	9.57 ± 0.36
sample 2	9.78 ± 0.28	10.16 ± 0.27
sample 3	9.82 ± 0.32	10.00 ± 0.25

## 4. Conclusion

A novel propofol detection method based on GQDs was established. GQDs were obtained through CA pyrolysis and characterized by UV–Vis absorption spectrum, fluorescence emission spectrum, FTIR spectroscopy and TEM. Under conditions of H_2_O_2_ and HRP, propofol could be oxidized to a product that could effectively quench GQDs fluorescence. A fluorescence quenching quantitative relationship between propofol oxidation products and GQDs was established. The fluorescence spectrophotometric method for the detection of propofol in the emulsion was developed and validated with HPLC. The developed approach is simple, non-toxic, environment-friendly and easy to perform. The developed approach not only provides a novel method for the detection of propofol in emulsion but also gives the new potential application of GQDs in pharmaceutical analysis.

## Data accessibility

This article does not contain any additional data.
